# The Clinical Usefulness of a Glaucoma Polygenic Risk Score in 4 Population-Based European Ancestry Cohorts

**DOI:** 10.1016/j.ophtha.2024.08.005

**Published:** 2024-08-14

**Authors:** Victor A. de Vries, Akiko Hanyuda, Joëlle E. Vergroesen, Ron Do, David S. Friedman, Peter Kraft, Constance Turman, Yuyang (Leo) Luo, Jessica H. Tran, Bart Liefers, Sze H. Wong, Rachel H. Lee, Nazlee Zebardast, Caroline C.W. Klaver, Ayellet V. Segrè, Louis R. Pasquale, Janey L. Wiggs, Jae H. Kang, Wishal D. Ramdas

**Affiliations:** 1 Department of Ophthalmology, Erasmus Medical Center, Rotterdam, The Netherlands.; 2 Department of Epidemiology, Erasmus Medical Center, Rotterdam, The Netherlands.; 3 Department of Ophthalmology, Keio University School of Medicine, Shinjuku-ku, Tokyo, Japan.; 4 Department of Genetics and Genomics Science, Icahn School of Medicine at Mount Sinai, New York, New York.; 5 Department of Ophthalmology, Massachusetts Eye and Ear, Harvard Medical School, Boston, Massachusetts.; 6 National Cancer Institute, National Institutes of Health, Bethesda, Maryland.; 7 Department of Epidemiology, Harvard School of Public Health, Boston, Massachusetts.; 8 Department of Ophthalmology, Icahn School of Medicine at Mount Sinai, New York, New York.; 9 Department of Ophthalmology, Radboud University Medical Center, Nijmegen, The Netherlands.; 10 Institute of Molecular and Clinical Ophthalmology, University of Basel, Basel, Switzerland.; 11 Channing Division of Network Medicine, Brigham and Women’s Hospital, Boston, Massachusetts.

**Keywords:** Genetic correlation, Genetic risk score, Intraocular pressure, Linkage disequilibrium score regression, Open-angle glaucoma

## Abstract

**Purpose:**

We used a polygenic risk score (PRS) to identify high-risk groups for primary open-angle glaucoma (POAG) within population-based cohorts.

**Design:**

Secondary analysis of 4 prospective population-based studies.

**Participants:**

We included four European-ancestry cohorts: the United States-based Nurses’ Health Study, Nurses’ Health Study 2, and the Health Professionals Follow-up Study and the Rotterdam Study (RS) in The Netherlands. The United States cohorts included female nurses and male health professionals ≤ 55 years of age. The RS included residents ≤ 45 years of age living in Rotterdam, The Netherlands.

**Methods:**

Polygenic risk score weights were estimated by applying the lassosum method on imputed genotype and phenotype data from the UK Biobank. This resulted in 144 020 variants, single nucleotide polymorphism and insertions or deletions, with nonzero βs that we used to calculate a PRS in the target populations. Using multivariable Cox proportional hazard models, we estimated the relationship between the standardized PRS and relative risk for POAG. Additionally, POAG prediction was tested by calculating these models’ concordance (Harrell’s C statistic). Finally, we assessed the association between PRS tertiles and glaucoma-related traits.

**Main Outcome Measures:**

The relative risk for POAG and Harrell’s C statistic.

**Results:**

Among 1046 patients and 38 809 control participants, the relative risk (95% confidence interval) for POAG for participants in the highest PRS quintile was 3.99 (3.08–5.18) times higher in the United States cohorts and 4.89 (2.93–8.17) times higher in the RS, compared with participants with median genetic risk (third quintile). Combining age, sex, intraocular pressure of more than 25 mmHg, and family history resulted in a meta-analyzed concordance of 0.75 (95% CI, 0.73–0.75). Adding the PRS to this model improved the concordance to 0.82 (95% CI, 0.80–0.84). In a meta-analysis of all cohorts, patients in the highest tertile showed a larger cup-to-disc ratio at diagnosis, by 0.10 (95% CI, 0.06–0.14), and a 2.07-fold increased risk of requiring glaucoma surgery (95% CI, 1.19–3.60).

**Conclusions:**

Incorporating a PRS into a POAG predictive model improves identification concordance from 0.75 up to 0.82, supporting its potential for guiding more cost-effective screening strategies.

**Financial Disclosure(s):**

Proprietary or commercial disclosure may be found in the Footnotes and Disclosures at the end of this article.

Glaucoma is a common neurodegenerative eye disease and the leading cause of irreversible blindness globally.^1^ Among the various forms of glaucoma, primary open-angle glaucoma (POAG) is the most prevalent in European populations, affecting approximately 1% to 3% of middle-aged to elderly individuals.^2^ Although no curative treatments exist for POAG, timely and appropriate interventions can slow disease progression effectively.^3–6^ Primary open-angle glaucoma is particularly insidious in its onset because it often leads to gradual visual field loss that goes unnoticed for extended periods. Around 50% of individuals with POAG in developed countries are without a diagnosis.^7^ Therefore, early screening initiatives are crucial for identifying cases, especially because early-stage treatment is more cost-effective.^8^ Unfortunately, population-based glaucoma screening programs have not proven cost-effective in developed nations because of the high implementation costs and the expenses related to false-positive referrals.^9,10^ Accordingly, identifying high-risk subgroups for glaucoma screening could represent a cost-effective strategy.

Primary open-angle glaucoma has a substantial hereditary component, with individuals having up to a 9-fold increased risk if they have a first-degree relative with the disease.^11^ Early investigations into the genetic architecture of POAG identified several critical Mendelian variants.^12^ Although these variants are relatively rare, they significantly impact POAG risk. Recently, genome-wide association studies (GWASs) of hundreds of thousands of individuals, including more than 34 000 patients with POAG, have identified hundreds of common genetic variants associated with POAG risk within the general population.^13^ Although the individual contribution of these variants to POAG risk is small, their combined effect can result in disease risk comparable with that of one of the high-penetrance Mendelian variants.^14^ Creating a polygenic risk score (PRS) by collectively aggregating the effects of these common variants has demonstrated the ability to capture much of the POAG heritability.^13^ To assess the potential of using genetic data for POAG prediction, we developed a PRS using a high-performing and computationally efficient technique called lassosum.^15^ This PRS then was applied to 4 prospective population-based cohort studies to estimate the extent of the increased risk associated with higher POAG PRS values. Additionally, we investigated the current potential of the PRS to predict POAG within these populations. Finally, we assessed the association between the PRS and glaucoma-related traits among patients in each cohort.

## Methods

### Study Design

We evaluated the usefulness of the POAG PRS in 4 prospective population-based cohorts: the Nurses’ Health Study, the Nurses’ Health Study 2, and Health Professionals Follow-up Study in the United States and the Rotterdam Study (RS) in The Netherlands. Detailed descriptions of these study populations have been published previously.^16,17^ Briefly, in the Nurses’ Health Study, 121 700 female nurses 30 to 55 years of age at enrollment answered the baseline questionnaire in 1976. The Nurses’ Health Study 2, with 116 429 female nurses 25 to 42 years of age, began in 1989. For our analyses, we combined data from these 3 United States–based cohorts and included only participants older than 55 years who had undergone eye examinations after the initial baseline survey. The Health Professionals Follow-up Study enrolled 51 529 male health professionals 40 to 75 years of age in 1986. Questionnaires related to lifestyle, diet, and health conditions, including glaucoma, were assessed biennially, with 80% or more response rates to follow-up questionnaires (Supplemental Methods A, available at www.aaojournal.org). The study protocol was approved by the institutional review boards of the Brigham and Women’s Hospital, the Harvard T.H. Chan School of Public Health, and the Icahn School of Medicine at Mount Sinai. All participants provided informed consent. This study adhered to the tenets of the Declaration of Helsinki.

The RS is a prospective population-based cohort study of people living in Ommoord, a district of Rotterdam, The Netherlands.^17^ The RS consists of 4 cohorts; however, because of the younger age at baseline and limited follow-up of the later cohorts, we limited our survival analyses to the first cohort, and only included previously patients with detected POAG from the second and third cohorts in the secondary within-case analysis (Supplemental Methods B, available at www.aaojournal.org). The first cohort (RS cohort I) started in 1990 and consisted of 7983 participants > 55 years of age (age range, 55.0–99.2; response rate, 78%). Follow-up examinations were performed every 4 to 5 years. Participants were examined for the presence of POAG at baseline and were invited for each follow-up round to be re-examined for the presence of POAG. Participants underwent an extensive physical examination at a research center as well as a home interview, including questionnaires on lifestyle factors and medication use (Supplemental Methods B). Overall, the glaucoma surveillance period was from 1980 through 2019 for the United States cohorts and from 1991 through 2019 in the RS. Participants were followed up until death, loss to follow-up, or the end of the study period.

The Medical Ethics Committee of Erasmus MC (identifier, MEC 02.1015) and the Dutch Ministry of Health, Welfare, and Sport (Population Screening Act WBO, license no. 1071272–159521-PG) approved the use of the individual-level data in RS participants. The RS is entered into the Dutch National Trial Register (www.onderzoekmetmensen.nl) and the World Health Organization International Clinical Trials Registry Platform (www.who.int/clinical-trials-registry-plotform) under shared catalog number NTR6831. All participants provided written informed consent following the Declaration of Helsinki to participate in the study and have their information obtained from their treating physicians.

### Genotyping, Imputation, and Polygenic Risk Score Development

For the United States cohorts, blood and saliva samples were collected and DNA was extracted.^18^ Multiple GWASs were conducted in nested case-control studies within the United States cohorts using 5 genotyping platforms: Affymetrix (Affymetix, Santa Clara, CA), HumanCoreExome (Twist Bioscience, San Francisco, CA), IlluminaHumanHap, OmniExpress, and OncoArray (Illumina, San Diego, CA).^18^ Genotype, imputation, and quality control of GWASs were standardized across the cohorts (Supplemental Methods A). For the RS, DNA extraction was performed using whole blood samples following standardized and previously described protocols.^17^ Genotyping was performed using both the Infinium II HumanHap550(-Duo) (RS cohorts I and II) and 610-Quad Genotyping BeadChip (Illumina; RS cohorts I and III; Supplemental Methods B). Imputation of markers was performed using the Trans-Omics for Precision Medicine reference panel for both the United States cohorts and the RS cohort. Only variants with high imputation quality scores (MachR^2^ / IMPUTE Info score > 0.3) were included in the analysis, and because of the relatively smaller sample size, rare variants (minor allele frequency < 0.01) for the RS also were excluded. We trained the PRS variant weights for POAG by applying the lassosum method,^15^ a penalized regression framework, to data of 449 186 cross-ancestry participants (14 171 patients and 435 015 control participants) in the UK Biobank. We leveraged GWAS summary statistics from a large cross-ancestry meta-analysis of POAG,^13^ excluding UK Biobank samples, as described by Singh et al.^19^ We included genotyped and imputed variants (MachR^2^ / IMPUTE Info score > 0.6) with a minor allele frequency of more than 0.01 (all variant calls were encoded as hard calls based on a posterior probability of > 0.9). Only the nonpseudoautosomal region on the X chromosome was included in all analyses, without Hardy-Weinberg equilibrium filtering. A total of 144 020 single nucleotide polymorphisms and insertions or deletions with nonzero βs were identified. We then applied these weights by multiplying them with the dosage values (the linear transformation of the posterior genotype probability of the effect allele), and the sum of these multiplications was considered the PRS for any given study participant. Dosage values for the X chromosome nonpseudoautosomal region were coded in male participants as (0, 1) and in female participants as (0, 1, 2).

### Primary Open-Angle Glaucoma Ascertainment

In the United States cohorts, patients with POAG were identified among participants who reported a new diagnosis of glaucoma on biennial questionnaires. We sought participant permission to access their relevant medical records and extracted confirmatory medical information, including maximum untreated intraocular pressure (IOP), optic nerve head structural information, glaucoma surgical history, and all available visual fields. If participants declined record access, they were excluded from all further analysis. A glaucoma specialist (L.R.P.) reviewed all the medical records to confirm a diagnosis of POAG by standardized criteria. In the United States cohorts, POAG confirmation required at least 2 reliable (≤ 20% for false-negative findings and false-positive findings and ≤ 33% for fixation loss rate) visual fields indicating reproducible defects consistent with glaucoma (Supplemental Methods A). In the RS, all participants were screened with a 52-point suprathreshold test using the Humphrey Field Analyzer (HFA II 740; Carl Zeiss). Details were published previously.^2^ Primary open-angle glaucoma was considered to be present if a defect was reproducible on both screening tests and the following 24–2 full-threshold or Swedish Interactive Thresholding Algorithm standard test, and no other cause could be identified (Supplemental Methods B).^20^ Within the RS, visual fields were considered reliable if the false-positive results and false-negative results were ≤ 33%, and fixation losses were ≤ 20%. For both the United States cohorts and the RS cohort, participants with other possible causes of visual field loss, signs of anterior chamber angle closure, or secondary glaucoma were excluded.

### Statistical Analyses

We calculated a standardized PRS for each participant (for the United States cohorts, standardization was carried out within each genotype platform separately) and estimated the relative risk with corresponding 95% confidence intervals (CIs) for POAG and each quintile of the PRS compared with the third quintile using multivariable Cox proportional hazard models. The third quartile was selected as the reference category because this group most accurately represents an average clinical patient. Moreover, this allowed us to compare all quintiles without the limitation of a small number of events in the group with the lowest PRS. For the United States cohorts, the event date for patients with POAG was defined as the earliest of the following: untreated IOP of > 21 mmHg, vertical cup-to-disc ratio (CDR) of ≥ 0.6, asymmetry in CDR of ≥ 0.2, or reproducible visual field loss. This information was extracted from medical records, in combination with questionnaires sent to treating eye care providers. For the RS, if POAG was ascertained at a visit, the event date was assumed to have been three-quarters of the way between that visit and the prior visit (because the risk would be higher toward the latter half of the interval, given the higher risk with increasing age). Finally, participants were censored at death (or the development of cancer for the United States cohorts), when lost to follow-up, or at the end of the study period. Model 1 was adjusted for age and sex. In model 2, we also adjusted for age and sex interaction and nonocular covariates, including hypertension, type 2 diabetes mellitus, body mass index (in kilograms per square meter), alcohol intake (grams daily), caffeine intake (grams daily), and total cholesterol levels (dichotomized as ≥ 240 mg/dl or < 240 mg/dl). Model 3 included the model 2 covariates with additional adjustment of IOP (dichotomized as yes or no IOP of > 25 mmHg), and model 4 incorporated the model 2 covariates with additional adjustment for family history of glaucoma. An inverse probability weighting was applied for the United States cohorts based on the probability of being selected for genotyping among those who provided a blood or saliva sample.^21^ We then calculated Harrell’s C statistic (i.e., concordance index), which can be interpreted as the approximate equivalent of an area under the receiver operating characteristic (ROC) curve (AUC) for time-dependent effects, using Cox proportional hazard models including age, sex, IOP, family history, and the POAG PRS. Finally, for the RS cohort, we calculated the C statistic for a model that also included retinal nerve fiber layer and ganglion cell layer complex thickness measures from OCT imaging. These variables were not measured at baseline, but halfway through follow-up (from September 2007 onward). Therefore, this model does not represent true prospective prediction, but we included it as an exploratory analysis to approximate the potential when including OCT parameters. Although the C statistic is the best representation of predictive discrimination for time-to-event data, it is a singular metric with no time-to-event equivalent of an ROC. Therefore, for illustrative purposes, we also performed logistic regression models and plotted the ROC to demonstrate the approximate sensitivity and specificity distribution of the PRS. In a case-only secondary analysis, we first evaluated the effect of the PRS on POAG-related traits (maximum IOP and CDR) in control participants to confirm that the PRS was behaving as expected. We then examined the association between PRS tertiles and various glaucoma-related traits (age at diagnosis, maximum IOP, CDR, mean deviation, and pattern standard deviation, and requirement of glaucoma surgery). Given the relatively small sample size and minimal evidence for confounding during the primary outcome analyses, associations of PRS with POAG-related traits were examined adjusting only for age and sex.

In both the primary (relative risk) and secondary within-case analyses, potential nonlinear relationships between PRS and the POAG-related traits were examined using restricted cubic spline analyses, with a *P* value of < 0.05 as the criterion for both inclusion and retention of the spline variables (i.e., knots) corresponding to a prespecified number of evenly spaced quantiles of the exposure distribution (e.g., PRS) in the model.^22^ Results were meta-analyzed using generic inverse variance weighted models, except for C statistics, which were logit-transformed and meta-analyzed using restricted maximum likelihood estimation.^23^ Heterogeneity of the meta-analyses was measured by calculating the *I*^2^ value. In case of low heterogeneity (*I*^2^ < 50%), a fixed-effects model was used in our meta-analyses. In case of high heterogeneity (*I*^2^ > 50%), a random-effects model was used. No *I*^2^ equivalent exists for the meta-analysis of C statistics, but because *I*^2^ was estimated at < 50% for the relative risks of all Cox hazard regression models (on which the C statistics were based), we assumed a fixed-effect model for these analyses.^24^ All significance tests were 2-sided, and a *P* value < 0.05 was considered statistically significant. Analyses were conducted with SAS version 9.4 software (SAS Institute), and R software versions 4.0.3 and 4.2.3 (R Foundation for Statistical Computing), and SPSS software version 28.0.1.0 for Windows (IBM, Inc.).

## Results

### Baseline Characteristics

For the United States cohorts, during 487 109 person-years of follow-up, 723 patients with incident POAG and 33 024 control participants were identified (Table 1). The mean ± standard deviation of the standardized POAG PRS was 0.8 ± 1.0 in the POAG group and 0.0 ± 1.0 in the control group. Within the RS cohorts, during 48 581 person-years of follow-up, 323 patients with incident POAG and 5785 control participants were included (Table 1). The mean ± standard deviation of the PRS was 1.0 ± 0.8 and 0.0 ± 1.0 within the patient and control groups, respectively. The PRS was normally distributed in both the United States cohorts and the RS cohort (Fig S1, available at www.aaojournal.org). Participants with higher PRS had more family members with POAG (Tables S2 and S3, available at www.aaojournal.org). The mean CDR among patients in both cohorts was 0.6, and the median mean deviation on the earliest available reliable visual field test was −4.5 dB and −6.2 dB in the United States and RS cohorts, respectively. As expected in both cohorts, compared with control participants, patients with POAG were more likely to have a first-degree family member with POAG or a maximum IOP > 25 mmHg.

### Relative Risk

**Figure 2.**
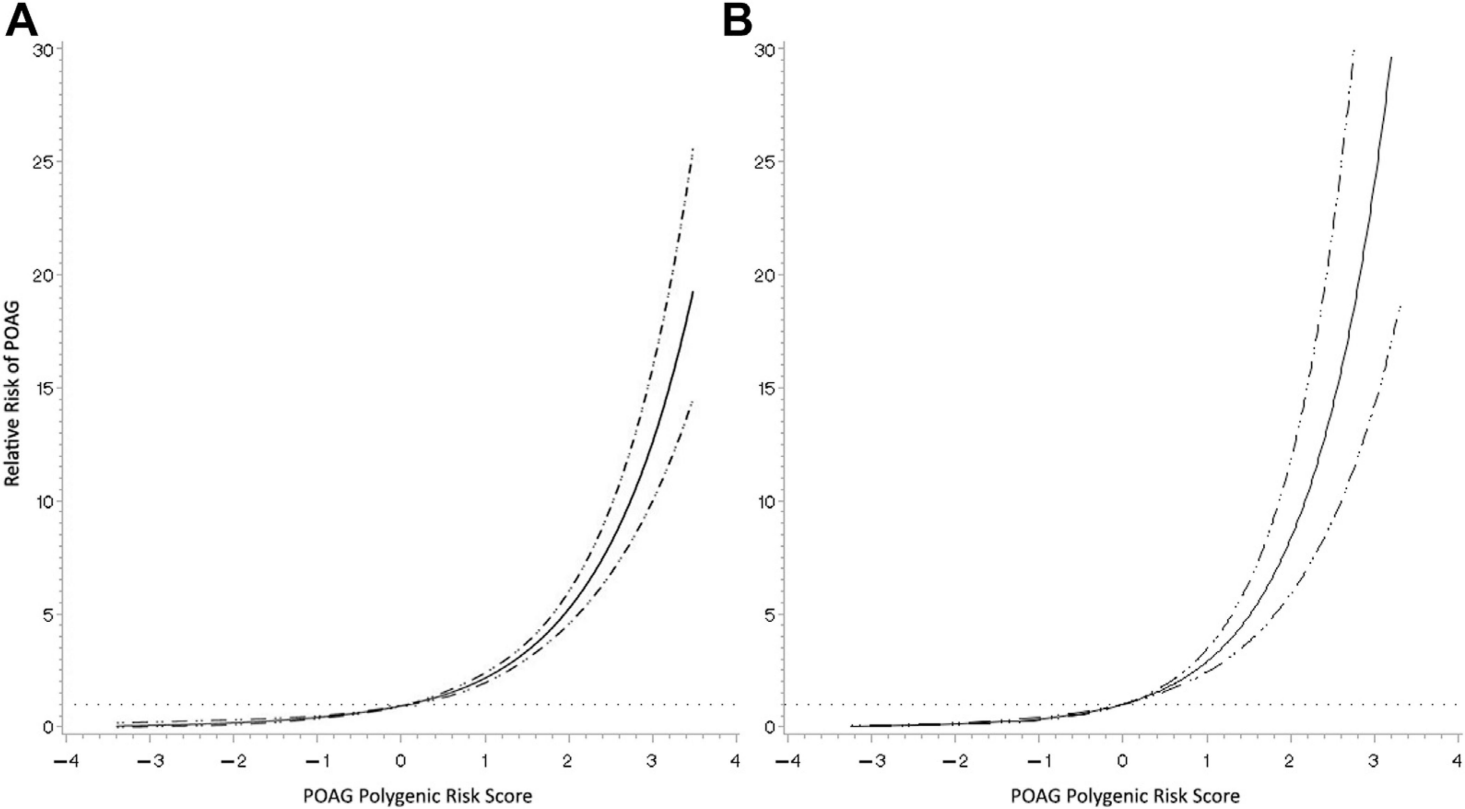
A, B, Relative risk with corresponding 95% confidence intervals (dash-dot lines) for each 1-standard deviation increase in polygenic risk score for (A) the United States cohorts and (B) Rotterdam Study (cohort I) compared with the primary open-angle glaucoma (POAG) risk of someone with a standardized polygenic risk score of 0. The dotted line represents a relative risk of 1.0.

The relative risk for POAG of participants in the highest PRS quintile was 3.99 (95% CI, 3.08−5.18) times higher compared with that of participants with median genetic risk (quintile 3) in the United States cohorts and 4.89 (95% CI, 2.93−8.17) times higher within the RS cohort (Table 4, model 1). The relative risk for an individual within the lowest quintile was 0.38 (95% CI, 0.24−0.60) times lower in the United States cohorts and 0.32 (95% CI, 0.13−0.80) times lower in the RS cohort compared with the third quintile (Table 4, model 1). In an analysis including only age and sex, comparing the top with the bottom quintile, the relative risk was 10.45 (95% CI, 6.97−15.68) in the United States cohorts and 16.31 (95% CI, 7.10−37.48) in the RS cohort. Relative risks were notably similar between the United States and RS cohorts. Estimated heterogeneity (*I*^2^) was < 50% for all models. The inclusion of nonocular covariates (i.e., model 2) did not affect the relative risk estimates substantially. The inclusion of IOP (i.e., model 3) or the inclusion of glaucoma family history (i.e., model 4) consistently attenuated the effect sizes. Relative risk plotted against the POAG PRS showed an exponential relationship in the United States cohorts and in the RS (*P* < 0.001 for spline; Fig 2).

### Primary Open-Angle Glaucoma Prediction

**Figure 3.**
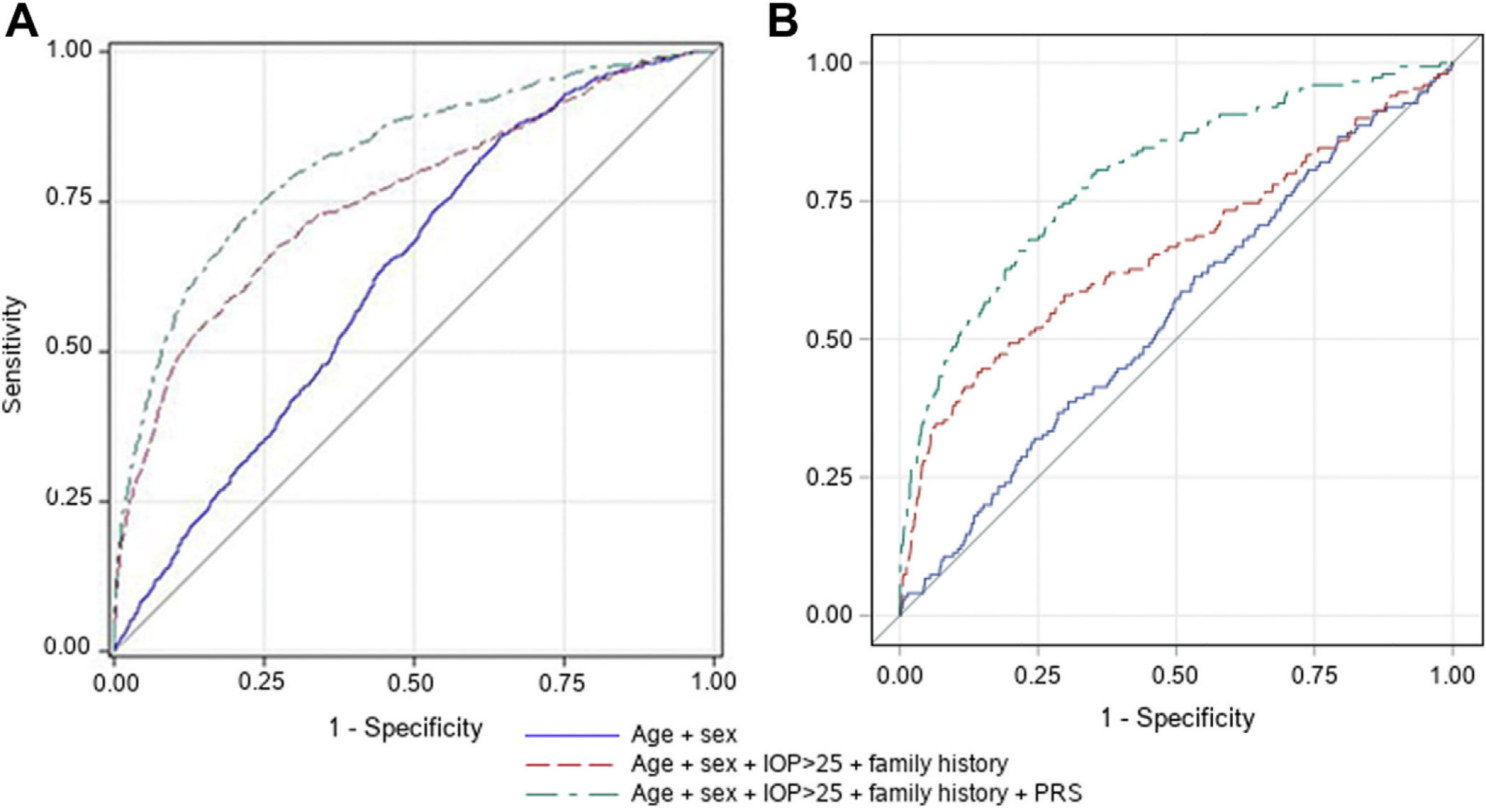
Receiver operating characteristic curves for primary open-angle glaucoma (POAG) based on the predicted probabilities from logistic regression models for (A) the United States cohorts and (B) the Rotterdam Study (cohort I). IOP = intraocular pressure; PRS = polygenic risk score. BMI = body mass index; IOP = intraocular pressure; MD = mean deviation; N/A = not available; PRS = polygenic risk score. Data are presented as number, no. (%), mean ± standard deviation, or median (interquartile range). *At the time of diagnosis.

Concordance in a Cox hazard regression model including age, sex, IOP, and family history was 0.75 (95% CI, 0.73−0.77) in the United States-based cohorts and 0.74 (95% CI, 0.69−0.79) in the RS cohort (Table 5). A model including the POAG PRS alone achieved a similar concordance of 0.74 (95% CI, 0.72−0.76) and 0.77 (95% CI−0.73, 0.81), respectively. Combining all the variables in a model resulted in a prediction concordance of 0.82 (95% CI, 0.80−0.84) in the United States cohorts and 0.82 (95% CI, 0.78−0.86) in the RS cohort. In comparison, the C statistic using panels of top genome-wide significant genetic markers from published meta-GWASs, also combined with the other covariates, was 0.78 (95% CI, 0.76−0.80) in the United States cohorts and 0.73 (95% CI, 0.69−0.78) in the RS cohort (Table S6, available at www.aaojournal.org), demonstrating the added value of also considering more modest effect sizes variants across the genome.^25^ Our exploratory analysis in the RS, including OCT parameters in the prediction model, resulted in an even better concordance of 0.87 (95% CI, 0.82–0.92; Table 5). Logistic regression models showed a similar AUC for most models (Table S7, available at www.aaojournal.org). The ROC based on these logistic regression models showed a gradual increase in specificity for the models including the PRS as the sensitivity threshold was relaxed (i.e., the ROCs were relatively symmetrical; Fig 3).

### Patient-Only Analyses

In the RS, the PRS was associated with increased IOP in control participants, with a β of 0.93 (95% CI, 0.81–1.1; *P* = 5.4 × 10^−52^). Similarly, the PRS was associated with an increased CDR in control participants, with a β of 0.04 (95% CI, 0.03–0.05; *P* = 7.6 × 10^−45^). Within the United States cohorts, patients with POAG in the highest PRS tertile on average were 1.53 years (95% CI, 0.14–2.91 years) younger when they received a diagnosis of POAG compared with the lowest tertile (*P* = 0.02 for trend; Table 8). No significant association was found between POAG PRS and the age at diagnosis within the RS cohorts (patients from RS cohorts I, II, and III combined). Within the RS cohorts, the maximum IOP for participants in the highest tertile was significantly higher, by 3.37 mmHg (95% CI, 0.88–5.86 mmHg) compared with the lowest tertile. In both the United States and the RS cohorts, participants in the highest tertile showed a statistically significant larger CDR at diagnosis, by 0.07 (95% CI, 0.03–0.12) and 0.22 (95% CI, 0.13–0.30), respectively. The association of POAG-related traits with PRS generally was linear (*P* > 0.05 for spline), except for a significant nonlinear relationship between glaucoma surgery requirement and PRS (*P* = 0.0003 for spline) in the United States cohorts, with an odds ratio for glaucoma surgery requirement of 2.38 (95% CI, 1.15–4.94) for the highest tertile of PRS. After meta-analyzing the results of both cohorts, only the increased CDR at diagnosis and an increased odds ratio for requiring glaucoma surgery remained statistically significant.

## Discussion

### Summary of the Results

In these prospective population-based cohort studies, we demonstrated an exponential increase in POAG risk as genetic predisposition increases. Including a POAG-specific PRS in a predictive model increased the concordance of POAG prediction from 0.75 to 0.82. A higher PRS also was associated with an increased requirement for glaucoma surgery.

### Relationship with the Literature

Primary open-angle glaucoma is an ideal target for screening efforts considering the clinical benefit of early detection, and both the costs and negative impact on quality of life and physical function associated with this late-stage blinding disease.^3^ Unfortunately, dedicated screening strategies currently are not cost-effective in high-income nations.^9,10^ Importantly, screening a population with a 2% POAG prevalence using a hypothetical test that has 80% sensitivity and 95% specificity yields a positive predictive value of only 25%. In a 2-tier detection strategy in which level 1 screening identifies participants in the top quintile of glaucoma PRS and applies the same hypothetical test only to these patients, the positive predictive value increases to up to 62% considering the 4.5-fold increase in POAG risk for people in the highest quintile relative to the third quintile of PRS, as described in our study. It would not be necessary to confine risk groups to the quintile cutoff points; given the exponential relationship between the PRS and POAG risk, the threshold for level 2 screening could be adjusted to suit the cost-benefit balance of any specific population. We acknowledge that 50% of patients with POAG would be missed if only the highest quintile was screened actively; nonetheless, being able to detect 50% of patients with POAG early and cost-effectively still would be a substantial public health gain. Additionally, patients with POAG in the highest PRS quintile showed a higher risk of requiring glaucoma surgery. Craig et al^26^ also showed in a clinical cohort that a high POAG PRS is associated with more rapid structural and functional POAG progression. We hypothesize that the 50% of patients with POAG captured in the highest quintile includes most patients who are at risk of end-stage disease developing and, therefore, would benefit the most from early detection.

Other studies also aimed to optimize PRS for risk assessment. Previously, Gao et al^27^ showed that relaxing the traditional GWAS significance threshold from *P* < 5 × 10^−8^ to *P* ≤ 5 × 10^−5^ and including the resulting 1691 IOP-associated single nucleotide polymorphisms in a PRS would result in an odds ratio for POAG of 6.34 (95% CI, 4.82–8.33) for the top quintile relative to the bottom quintile.^27^ With this PRS, the AUC for POAG based on logistic regression was 0.77. A recent multitrait analysis for POAG found an odds ratio of 14.9 (95% CI, 10.7–20.9) for the highest versus the lowest decile and an AUC of up to 0.80.^26^ In comparison, in our analyses, we observed up to a 16.3-fold increase in relative risk when comparing the top quintile with the bottom quintile and a maximum C statistic of 0.82 when including demographic variables along with the PRS. When comparing the effectiveness of the PRS of Han et al^25^ directly with the present cohorts, the meta-analyzed C statistic was 0.78 (95% CI, 0.76–0.79) compared with 0.82 (95% CI, 0.80–0.84) in our PRS (Table S6). This suggests that a PRS encompassing variants across the genome, including those with more modest effects, using the lassosum method captures more POAG heritability than the traditional genetic risk scores that consider only the genome-wide significant variants.

We found that our POAG PRS also was associated with increased CDR at the time of diagnosis. Although other associations for disease severity were not significant consistently between both cohorts, all were in the expected direction when comparing participants in the third tertile versus those in the first tertile. Notably, a recent study by Marshall et al^28^ showed that patients with untreated suspected glaucoma within the highest quintile of a POAG PRS were up to 3.3 times more likely to start therapy compared with the bottom quintile, whereas those already receiving therapy at enrollment were 1.8 times more likely to require an escalation of therapy. These findings suggest that glaucoma PRS can be a valuable metric for predicting POAG progression in both those at risk of POAG and patients with POAG.

### Strengths and Limitations

First, the greatest strength of our study is its population-based design, allowing us to estimate the relationship between genetic predisposition and POAG in the general population. Our results are broadly generalizable in European populations, because our findings are consistent between the United States cohorts and in RS participants. Second, most similar studies primarily rely on large publicly available datasets from biobanks or public health care registries using self-reported POAG as the primary outcome. Unconfirmed self-reported glaucoma status is known to be an imprecise measure of POAG incidence,^29,30^ especially because those without glaucoma are prone to misclassification because true cases of glaucoma are not always diagnosed. Therefore, our outcome measures are robust measures of true POAG risk in a general population of European ancestry. Third, instead of traditional *P* value thresholds and linkage disequilibrium pruning, we used machine learning in the form of the lassosum method for variant selection. This, in combination with the inclusion of X chromosome variants in our analyses, likely allowed us to capture more of POAG heritability. We show that this approach modestly increases POAG prediction and that the resulting PRS is the best-performing genetic screening tool in our cohorts. Finally, our study design allowed us to estimate the relationship between key parameters of interest such as age at diagnosis.

However, some limitations should be considered as well. First, the robust performance of the glaucoma PRS could be related partially to the fact that the populations studied contributed to the PRS formation; however, the same PRS also has shown usefulness in populations not included in generating the glaucoma PRS, including populations with non-European people. For example, the same glaucoma PRS showed an AUC of 0.81 and 0.77 for Europeans and for Africans, respectively, in BioMe, a diverse biorepository linked to an inner New York City hospital system.^31^ Nonetheless, the present population-based cohorts were almost exclusively of European ancestry, and we could not examine the performance of our PRS in non-European populations. Although our PRS was developed on cross-ancestry GWAS data, participants of European ancestry remain overrepresented in the literature, and the extent to which our PRS can be generalized to other populations requires continued exploration. Second, our study populations were undergoing eye examinations regularly; thus, this may limit the generalizability of our findings to less frequently screened populations. Third, it is possible that our secondary within-case analyses would show a more consistent trend toward increased disease severity at the upper end of the PRS spectrum if we could stratify these analyses further (i.e., quartiles, quintiles, or even deciles). Unfortunately, the number of cases in the lower tertiles or quintiles restricted our analyses. Alternatively, a machine learning approach trained on a dichotomous outcome might bring optimal prediction performance for disease incidence but would be less useful compared with a multitrait genome-wide approach to predict associated glaucoma features. Fourth, our meta-analysis included only 4 cohorts. Estimation of combined effects, and especially heterogeneity, can be suboptimal when including a small number of studies in the meta-analysis. Finally, we did not investigate the interaction of our PRS with pathogenic *MYOC* gene variants.

## Conclusions and Future Considerations

Our study demonstrated the value of genetic risk assessment in the general population. Prediction concordance of a POAG PRS, and therefore clinical usefulness, is expected to increase over time as both the availability of genetic data and the sophistication of statistical and machine learning techniques improve. Cost-benefit analyses should be performed by combining the benefits of the many genetically predisposed conditions that may be predicted in part to estimate whether population-based genetic screening could become viable in the future.

## Supplementary Material

complete list of collaboratos

Suppl F1

Suppl F2

Table S2

Table S3

suppl appendix

Table S6

Table S7

## Figures and Tables

**Table 1.** Baseline Characteristics

	United States Cohorts		Rotterdam Study Cohorts	
		
Trait	*Patients*	*Control Participants*	*Patients*	*Participants*
No. of participants	723	33 024	323	5785
Female participants	478 (66.1)	23 447 (71.0)	157 (48.6)	3468 (59.9)
Age at enrollment (yrs)	58.2 ± 4.4	57.5 ± 3.9	68.4 ± 8.3	69.1 ± 8.9
Maximum IOP > 25 mmHg	85 (11.8)	217 (0.7)	126 (39.0)	334 (5.8)
Cup-to-disc ratio*	0.6 ± 0.2	N/A	0.6 ± 0.2	0.3 ± 0.2
Median visual field MD, dB*	−4.5 (−7.1 to −2.4)	N/A	−6.2 (−3.5 to −9.3)	N/A
Standardized glaucoma PRS	0.8 ± 1.0	0.0 ± 1.0	1.0 ± 0.8	0.0 ± 1.0
Family history of glaucoma	200 (27.7)	5105 (15.5)	44 (13.6)	398 (6.9)
Hypertension	182 (25.2)	10 098 (30.6)	197 (61.0)	3565 (61.6)
Diabetes mellitus	29 (4.0)	1754 (5.3)	15 (4.6)	342 (5.9)
BMI (kg/m^2^)	24.8 ± 3.8	25.5 ± 4.4	25.9 ± 3.2	26.3 ± 3.7
Blood pressure (mmHg)				
Systolic	128.5 ± 10.3	125.6 ± 11.6	139.2 ± 21.4	139.2 ± 22.5
Diastolic	79.8 ± 7.3	78.0 ± 7.5	74.2 ± 12.3	73.8 ± 11.6
Cigarette smoking (pack-years)	1.0 (0.0–20.0)	0.0 (0.0–15.0)	4.6 (0.0–27.0)	4.0 (0.0–26.4)
Caffeine intake (mg/day)	316.9 ± 235.1	275.1 ± 216.7	240.2 ± 133.9	224.7 ± 119.8
Alcohol intake (g/day)	8.1 ± 11.2	7.6 ± 11.4	11.3 ± 16.6	10.3 ± 14.8
Total serum cholesterol level (mg/dl)	207.4 ± 33.1	200.1 ± 34.4	245.6 ± 51.2	255.2 ± 47.1
Serum cholesterol ≥ 240 mg/dL	176 (24.3)	12 348 (37.4)	195 (60.4)	3707 (64.1)

**Table 4.** Multivariable-Adjusted Relative Risks with Corresponding 95% Confidence Intervals for Each Quintile of the Polygenic Risk Score, with the Third Quintile as the Reference Category

	Quintile				
	
Variable	*1*	*2*	*3*	*4*	*5*
US cohorts No. of events	44	63	101	153	362
PRS, median (IQR)	−1.29 (−4.13 to −0.84)	−0.54 (−0.84 to −0.27)	−0.01 (−0.27 to 0.24)	0.51 (0.24–0.82)	1.25 (0.82–4.79)
Model 1*	0.38 (0.24–0.60)	0.68 (0.47–0.98)	1.0	1.45 (1.07–1.97)	3.99 (3.08–5.18)
Model 2†	0.38 (0.24–0.60)	0.69 (0.47–0.99)	1.0	1.46 (1.08–1.98)	4.03 (3.11–5.23)
Model 3‡	0.40 (0.25–0.65)	0.69 (0.48–1.00)	1.0	1.41 (1.04–1.92)	3.86 (2.96–5.02)
Model 4§	0.39 (0.25–0.62)	0.71 (0.49–1.02)	1.0	1.50 (1.12–2.03)	3.89 (3.00–5.05)
RS-I					
No. of events	6	12	18	35	79
PRS, median (IQR)	−1.28 (−4.17 to −0.85)	−0.53 (−0.85 to −0.27)	−0.01 (−0.27 to 0.25)	0.52 (0.26–0.83)	1.29 (0.84–3.76)
Model 1	0.32 (0.13–0.80)	0.68 (0.33–1.41)	1.0	1.81 (1.03–3.21)	4.89 (2.93–8.17)
Model 2	0.27 (0.10–0.70)	0.62 (0.30–1.30)	1.0	1.75 (0.99–3.11)	4.90 (2.93–8.20)
Model 3	0.29 (0.11–0.77)	0.62 (0.30–1.30)	1.0	1.64 (0.92–2.92)	4.45 (2.66–7.46)
Model 4	0.26 (0.10–0.69)	0.62 (0.30–1.31)	1.0	1.75 (0.99–3.12)	4.71 (2.81–7.88)
Meta-analysis					
No. of events	50	75	119	188	441
Model 1	0.37 (0.24–0.55)	0.68 (0.49–0.94)	1.0	1.52 (1.16–1.99)	4.16 (3.30–5.24)
Model 2	0.36 (0.24–0.54)	0.68 (0.48–0.94)	1.0	1.52 (1.16–1.99)	4.19 (3.33–5.29)
Model 3	0.38 (0.24–0.58)	0.68 (0.49–0.94)	1.0	1.46 (1.11–1.91)	3.98 (3.14–5.03)
Model 4	0.36 (0.24–0.55)	0.69 (0.50–0.96)	1.0	1.55 (1.19–2.02)	4.04 (3.21–5.10)

IQR = interquartile range; PRS = polygenic risk score; RS = Rotterdam Study.

* Adjusted for age and sex.

† Adjusted for age, sex, age × sex, hypertension, type 2 diabetes, body mass index, alcohol and caffeine consumption, and total serum cholesterol level.

‡ Model 2 plus intraocular pressure.

§ Model 2 plus family history of glaucoma.

**Table 5.** Harrell’s C Statistic (Concordance) with Corresponding 95% Confidence Intervals Based on Cox Proportional Hazard Regression Models

Variable	C Statistic		

	*United States Cohorts*	*Rotterdam Study Cohort I*	*Meta-analysis*
Age plus sex	0.63 (0.61–0.64)	0.67 (0.62–0,72)	0.63 (0.62–0.65)
Age plus sex plus IOP > 25 mmHg	0.73 (0.71–0.75)	0.74 (0.69–0.79)	0.73 (0.71–0.75)
Age plus sex plus IOP > 25 mmHg plus family history	0.75 (0.73–0.77)	0.74 (0.69–0.79)	0.75 (0.73–0.75)
Age plus sex plus IOP > 25 mmHg plus family history plus PRS	0.82 (0.80–0.84)	0.82 (0.78–0.86)	0.82 (0.80–0.84)
PRS only	0.74 (0.72–0.76)	0.77 (0.73–0.81)	0.75 (0.73–0.77)
Age plus sex plus IOP > 25 mmHg plus family history plus PRS plus RNFL plus GCC thickness		0.87 (0.82–0.92)	

GCC = ganglion cell complex; IOP = intraocular pressure; PRS = polygenic risk score; RNFL = retinal nerve fiber layer.

**Table 8.** Linear and Logistic Regression Models for Differences in Age at Diagnosis and Primary Open-Angle Glaucoma Severity Measures, Adjusted for Age and Sex

	Tertile		
	
Variable	*1*	*2*	*3*
United States cohorts No. of patients	89	164	470
Age at diagnosis (yrs)^†^	β = 0.0	−0.83 (−2.40 to 0.75)	−1.53 (−2.91 to −0.14)*
Maximum IOP (mmHg)	β = 0.0	0.37 (−1.00 to 1.73)	0.35 (−0.85 to 1.55)
CDR at diagnosis	β = 0.0	0.05 (−0.003 to 0.10)	0.07 (0.03–0.12)*
Visual field (dB) MD	β = 0.0	−0.74 (−2.01 to 0.54)	−0.97 (−2.10 to 0.16)
PSD	β = 0.0	0.14 (−0.75 to 1.03)	0.66 (−0.13 to 1.45)
Requirement of glaucoma surgery	OR = 1.0	OR = 2.40 (1.09–5.27)*	OR = 2.38 (1.15–4.94)*
RS cohorts No. of patients	35	83	205
Age at diagnosis (yrs)^†^	β = 0.0	−0.21 (−2.81 to 2.39)	0.49 (−1.96 to 2.93)
Maximum IOP (mmHg)	β = 0.0	1.38 (−1.78 to 4.54)	3.37 (0.88–5.86)*
CDR at diagnosis	β = 0.0	0.09 (−0.01 to 0.19)	0.22 (0.13–0.30)*
Visual field (dB) MD	β = 0.0	−0.02 (−2.04 to 2.00)	−1.23 (−3.54 to 1.08)
PSD	β = 0.0	0.63 (−0.65 to 1.90)	1.13 (−0.12 to 2.39)
Requirement of glaucoma surgery	OR = 1.0	OR = 0.88 (0.34–2.29)	OR = 1.71 (0.73–4.00)
Meta-analysis No. of patients	124	247	675
Age at diagnosis (yrs)^†^	β = 0.0	−0.66 (−2.01 to 0.68)	−1.04 (−2.24 to 0.17)
Maximum IOP (mmHg)	β = 0.0	0.53 (−0.72 to 1.78)	0.92 (−0.16 to 2.00)
CDR at diagnosis	β = 0.0	0.06 (0.01–0.10)*	0.10 (0.06–0.14)*
Visual field (dB) MD	β = 0.0	−0.53 (−1.61 to 0.54)	−1.02 (−2.04 to −0.01)*
PSD	β = 0.0	0.30 (−0.43 to 1.03)	0.79 (0.12–1.46)*
Requirement of glaucoma surgery	OR = 1.0	OR = 1.51 (0.87–2.93)	OR = 2.07 (1.19–3.60)*

CDR = cup-to-disc ratio; IOP = intraocular pressure; MD = mean deviation; OR = odds ratio; POAG = primary open-angle glaucoma; PSD = pattern standard deviation; RS = Rotterdam Study.

Data are expressed as β or OR with corresponding 95% confidence interval in parentheses.

* *P* < 0.05.

† Adjusted for age at enrollment.

## Abbreviations and Acronyms:

AUC: area under the receiver operating characteristic curve
BMI: body-mass index
CDR: cup-to-disc ratio
CI: confidence interval
GCC: ganglion cell complex
GWAS: genome-wide association study
MD: mean deviation
IOP: intraocular pressure
POAG: primary open-angle glaucoma
PRS: polygenic risk score
PSD: pattern standard deviation
RNFL: retinal nerve fiber layer
ROC: receiver operating characteristic
RS: Rotterdam Study
