# Supplementary material for: The Clinical Usefulness of a Glaucoma Polygenic Risk Score in 4 Population-Based European Ancestry Cohorts
Source: Ophthalmology. Author manuscript; Available in PMC 2025 Jun 27. (PMC12204775; doi:10.1016/j.ophtha.2024.08.005)

Supplementary Figure S1. Density plots for the primary open angle glaucoma (POAG) polygenic risk score for the US cohorts (A) and the Rotterdam Study (RS-I, B)

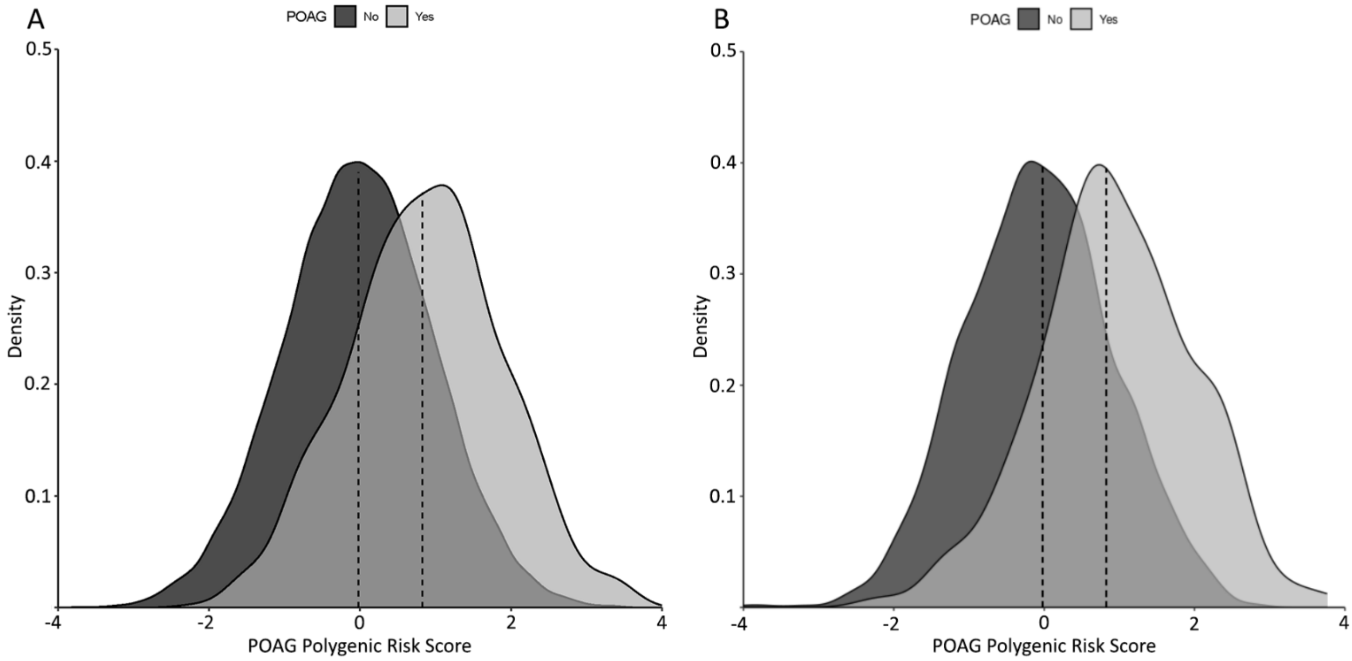

Supplement: Suppl F2 [file NIHMS2083589-supplement-Suppl_F2.pdf]
