# Supplementary material for: The Clinical Usefulness of a Glaucoma Polygenic Risk Score in 4 Population-Based European Ancestry Cohorts
Source: Ophthalmology. Author manuscript; Available in PMC 2025 Jun 27. (PMC12204775; doi:10.1016/j.ophtha.2024.08.005)
Supplement: Table S2 [file NIHMS2083589-supplement-Table_S2.pdf]

Supplementary Table S3. Mean difference in standardized polygenic risk score for participants with different kinds of affected family members with primary open-angle glaucoma, with corresponding 95% confidence intervals (age and sex adjusted)

	Beta (US cohorts)	Beta (RS-I)	Beta (meta-analysis)
Per family member (unspecified) with POAG	0.19 (0.16, 0.21)<br>N = 5305	0.28 (0.17, 0.39)<br>N = 354	0.22 (0.14, 0.30)<br>N = 5659
Per parent(s) with POAG	0.20 (0.17, 0.23)<br>N = 4120	0.23 (0.08, 0.38)<br>N = 184	0.20 (0.17, 0.23)<br>N = 4304
Per sibling(s) with POAG	0.28 (0.21, 0.35)<br>N = 806	0.35 (0.17, 0.52)<br>N = 157	0.28 (0.22, 0.35)<br>N = 963
Per child(ren) with POAG	0.02 (-0.38, 0.42)<br>N = 24	0.60 (0.01, 1.18)<br>N = 13	0.27 (-0.30, 0.83)<br>N = 37

N = the number of family members with a positive history of glaucoma (i.e. the number of events); US = United States; RS = Rotterdam Study
