# Supplementary material for: The Clinical Usefulness of a Glaucoma Polygenic Risk Score in 4 Population-Based European Ancestry Cohorts
Source: Ophthalmology. Author manuscript; Available in PMC 2025 Jun 27. (PMC12204775; doi:10.1016/j.ophtha.2024.08.005)
Supplement: suppl appendix [file NIHMS2083589-supplement-suppl_appendix.pdf]

Supplementary appendix

The clinical utility of a glaucoma polygenic risk score in four population-based European-ancestry cohorts

Contents

Supplementary Methods A: Study population, genotyping and imputation, ophthalmic assessment, and covariate definitions in the Nurses' Health Study (NHS), Health Professionals Follow-up Study (HPFS), and NHS2.

Supplementary Methods B: Study population, genotyping and imputation, ophthalmic assessment, and covariate definitions in the Rotterdam Study

Supplementary references

Supplementary Table S2: Mean difference in standardized polygenic risk score of participants with an increasing number of family members with primary open-angle glaucoma, with corresponding 95% confidence intervals

Supplementary Table S3: Mean difference in standardized polygenic risk score for participants with different kinds of affected family members with primary open-angle glaucoma, with corresponding 95% confidence intervals

Supplementary Table S6: Comparison of the concordance (Harrell's C-statistic) of our polygenic risk score and previously published genetic risk scores, with corresponding 95% confidence intervals. Age and sex were included in all models.

Supplementary Table S7: Area under the receiver operating characteristic curve (AUC) with corresponding 95% confidence intervals based on predicted probabilities from logistic regression models

Supplementary Figure S1: Density plots for the primary open-angle glaucoma (POAG) polygenic risk score for the US cohorts (A) and the Rotterdam Study (B)

Supplementary Methods A

The Nurses' Health Study (NHS), Health Professionals Follow-up Study (HPFS), and NHS2.

Study population

In the NHS, 121,700 female nurses aged 30-55 years at enrollment answered the baseline questionnaire in 1976. The HPFS enrolled 51,529 male health professionals aged 40-75 years in 1986. The NHS2, with 116,429 female nurses aged 25-42 years, began in 1989. In NHS, 32,826 women provided blood samples during 1989-1990 and 48,053 women provided saliva samples in 2001-2004. In NHS2, 29,611 women provided blood samples in 1996-1999, and 29,700 women provided saliva samples in 2004-2006. In HPFS, 18,159 blood samples were collected in 1993-1995, and 18,443 saliva samples were collected in 2004-2006. Samples were shipped by overnight courier, and sample processing was done at the laboratory.

Baseline was defined as the first two-year risk period that an eligible participant contributed person-time. In the prospective analyses to evaluate the relation between the POAG PRS and incident POAG, we followed and accrued person-time from eligible participants during 1980-2018 in NHS, 1989-2019 in NHS2, and 1986-2018 in HPFS. Among 289,658 participants across the 3 cohorts, we excluded 128,202 who did not provide a biospecimen, 119,067 without genotyping data, 2,848 participants who had incomplete baseline questionnaire data, 387 had prevalent cancer (except non-melanoma skin cancer), 445 had prevalent glaucoma, 3 who were lost to follow-up within 2 years after baseline questionnaire return, 805 who never reported an eye exam during follow-up, 190 were of African or Asian ancestry and 3970 died or were lost to follow-up before reaching age 55, leaving 33,747 eligible participants. At each 2-year follow-up period, we included only those who were >55 years old and reported an eye exam in the risk period. The vital status and date of death was confirmed from reports from the US Postal Service (when biennial questionnaires are sent to participants) and next-

of-kin, supplemented with annual National Death Index searches[1].

Ascertainment of POAG

POAG cases were identified among participants who reported a new diagnosis of glaucoma on biennial questionnaires. We requested permission from participants to access their medical records related to their glaucoma diagnosis from their eye care provider and sought confirmatory medical information including maximal untreated intraocular pressure (IOP), gonioscopy findings, optic nerve head structural information, glaucoma surgical history, and all available visual fields. If participants refused medical record access, they were excluded from all further analysis. A glaucoma specialist (LRP) reviewed all the medical records to confirm a diagnosis of POAG by standard criteria. POAG confirmation required the following: (a) at least two reliable (≤20% for false negative rate and false positive rate and ≤33% for fixation loss rate) visual fields indicating reproducible defects consistent with glaucoma, (b) non-occludable angles in both eyes by gonioscopy or slit lamp, and (c) absence of signs consistent with secondary glaucoma (e.g., pigment dispersion syndrome, trauma, uveitis, exfoliation syndrome, or rubeosis iridis).

Assessment of covariates

Information on covariates (i.e., body mass index, history of hypertension, type 2 diabetes mellitus, cholesterol levels) was collected with biennial self-reported questionnaires. Dietary information (i.e., caffeine and alcohol intake) was collected using validated Food Frequency Questionnaires. Whether a participant was ever noted to have IOP ≥25 mm Hg in either eye was asked in 1996 and every 4 years thereafter until 2012 for NHS and in 2008 for HPFS (not asked for NHS2). We collected information on family history of glaucoma (in biological parents or siblings) in 2000 for both NHS and HPFS and in 2017 for NHS2.

Genomic imputation and quality control

The genomic datasets, imputation, and quality control procedures were conducted separately in each population and were described with details in previous publications[1-2]. Briefly, the participants from five sub-populations were genotyped at different times and by different genotyping platforms. The subjects in “Affymetrix” were genotyped by the Genome-wide Human SNP Array 6.0. The subjects in “Illumina” were genotyped by either Illumina HumanHap300 BeadChip, HumanHap550-Quad BeadChip, Human610-Quad BeadChip, or Human660W-Quad BeadChip. The subjects in “OmniExpress” were genotyped by Illumina HumanOmniExpress-12 BeadChip. The subjects in “OncoArray” were genotyped by Infinium OncoArray-550K BeadChip. The subjects in “HumanCore” were genotyped by Illumina HumanCoreExome-12v1-0 BeadChip.

Variants with low call rate (<95%) were removed. A pairwise identity-by-descent analysis was conducted to identify duplicates. For individuals who were genotyped more than once using different genotyping platforms, one sample of the duplicated pair was excluded by the order of “Affymetrix,” “Illumina,” “OmniExpress,” “OncoArray,” and “HumanCore.” For individuals with different cohort IDs but a high genetic concordance rate, both of the pairs were removed. Genome imputation was further conducted in each population using the 1000 Genomes Project Phase 3 Integrated Release Version 5 as reference panels. Software ShapeIT (v2.r837) was used for genotype phasing, and the phased genotypes were further imputed to ~47 million variants with the TOPMed reference panel using Minimac3[3-4].

Supplementary Methods B

The Rotterdam Study

Study population

The Rotterdam Study (RS) is a prospective population-based cohort study of people living in Ommoord, a district of the city of Rotterdam[5]. The RS consists of three cohorts and is currently in the data collection stage of a fourth cohort. However, due to the younger age at baseline and limited follow-up of the later cohorts, we limited our survival analyses to the first cohort (RS-I), and only included the already detected POAG-cases of the second and third cohorts (RS-II, RS-III) in the secondary within-case analysis. The first cohort (RS-I) started in 1990 and consisted of 7,983 participants >55 years old (range 55.0 – 99.2, response rate of 78%). The second cohort (RS-II) started recruiting in 2000 and 3,011 participants >55 years old (range 55.2 – 98.9) were included (response rate of 67.3%). The third cohort (RS-III) also included people aged >45 years old (range 45.7 - 90.1) and consisted of 3,932 participants (response rate 64.9%) starting from the year 2006. Follow-up examinations were performed between 1993-1995 (no open-angle glaucoma (OAG) assessments), 1997-1999, 2002-2004, 2009-2011, 2014-2015, and 2018-2019 for RS-I. Similarly, participants from RS-II were evaluated between, 2004-2005, 2011-2012, and 2015-2016. Finally, follow-up examinations were performed between 2012-2014 for RS-III. Considering all three cohorts of RS combined 14,921 participants were included in this study, of which 11,496 were genotyped.

Genotyping and imputation

DNA extraction was performed using whole blood samples following standardized and previously described protocols[5]. Genotyping was performed using both the Infinium II HumanHap550(-Duo) (RS-I & RS-II) and 610-Quad Genotyping BeadChip (RS-I & RS-III; Illumina, San Diego, CA, USA). Imputation of markers was performed using the TOPMed reference panel. RS-I, RS-II, and RS-III were imputed separately on the Michigan imputation server. The following exclusions were applied to identify a

final set of SNPs that were used in this study: MAF <0.05, SNP call rate <0.95, and/or HWE p-value $<1x10^{-7}$. Additionally, markers with poor imputation quality scores (R^2<0.3) were removed.

Ophthalmic assessment

Participants were examined for the presence of OAG at baseline and were invited for each follow-up round to be re-examined for the presence of OAG. All participants underwent visual field testing using the Humphrey Field Analyzer (HFA II 740; Carl Zeiss, Oberkochen, Germany). Both eyes were screened using a 52-point supra-threshold test. If a participant did not respond to the light stimulus for three or more test locations, or four when the defect included the blind spot, the screening test was repeated. At the most recent follow-up visit of the first cohort (RS-I-7) participants were instead tested with a 17-point 20° supra-threshold test using Humphrey Frequency Doubling Technology (FDT 710; Carl Zeiss, Oberkochen, Germany). This test was repeated if at least one stimulus was missed at the $p < 0.05$ threshold level. If a defect could be reproduced (at least one overlapping test point was missed), a HFA 24-2 full-threshold (RS-II-1, RS-III-1) or 24-2 or Swedish Interactive Thresholding Algorithm (SITA) standard test (RS-II-2, RS-II-3, RS-II-4, and RS-III-2) visual field test was performed[6]. This test was classified as abnormal if at least one of three criteria was met: (1) the Glaucoma Hemifield Test was 'outside normal limits', (2) a minimum of three contiguous points in the pattern deviation probability plot with a reduction in sensitivity with a $p < 0.05$, of which at least one point with a $p < 0.01$, or (3) an overall Pattern Standard Deviation of $p < 0.05$. Visual field loss was considered to be present if the visual field tests were reliably performed and a defect was reproducible on both screening tests and the extensive test. Defects had to be in the same hemifield and at least one depressed test point had to have the same location on all tests. Visual field tests were considered reliable if the false positives and false negatives were < 33% and fixation losses were < 20%. If no other cause could be identified, and no homonymous defects or recognizable patterns like rim artifacts were observed, the defect was considered glaucomatous visual field loss (GVFL). Hospital records were retrieved for all participants with GVFL. Participants with other

possible causes of visual field loss, signs of anterior chamber angle closure, or secondary glaucoma, were excluded. If a defect was consistently reproduced without other possible causes for visual field loss, but our visual field tests were unreliable, hospital records were retrieved and the treating physician's diagnosis regarding the etiology of the defect was considered final. POAG was defined as GVFL in at least one eye, independent of IOP. IOP was measured using Goldmann applanation tonometry at baseline (Haag-Streit AG, Bern, Switzerland). Three measurements were taken from each eye, of which the median value was recorded. Untreated IOP levels were not recorded for participants receiving IOP-lowering medication. Therefore, we imputed the IOP for these participants. As an average reduction in IOP of approximately 30% has been reported in previous meta-analyses we divided the measured IOP by 0.7 to estimate the untreated IOP, and the IOP of participants who had undergone glaucoma surgery before baseline IOP measurement was set to 30 mmHg[7-8]. For vertical cup-disc ratio (CDR) measurements, simultaneous stereo color photos of the optic nerve head were taken at a fixed angle of 20° and analyzed with a computerized image analyzer (Topcon ImageNet System; ImageNet, Topcon Corporation, Tokyo, Japan) during baseline and the first follow-up visits with glaucoma assessment (RS-I-1, RS-I-3, and RS-II-1). For RS-III and later follow-up rounds of RS-I and RS-II, the Heidelberg Retina Tomograph (HRT; Heidelberg Engineering, Dossenheim, Germany) was used to calculate the linear cup-disc ratio (lCDR), which was used as an equivalent outcome for the CDR analyses. OCT-images were obtained from September 2007 (RS-I-5, RS-II-3, and RS-III-1) onwards, using the 3D macular 512x128 scan mode (scan area of 6.0x6.0mm) of the Topcon 3D-OCT 1000 mk2 (Topcon, Tokyo, Japan) or the Topcon 3D-OCT 2000 (Topcon, Tokyo, Japan). Using an in-house deep learning model that relies on a convolutional neural network, the average thickness of the retinal nerve fiber layer (RNFL), the ganglion cell layer (GCL), and the inner plexiform layer (IPL) for each B-scan was obtained. For model training, a total of 419 semi-automatically segmented B-scans from 152 distinct OCT volumes sourced from the RS were utilized in a cross-validation setting. The average differences between the model's predictions and the graders' annotations were smaller than the average differences observed between the graders

themselves (based on both the Dice coefficient and mean squared error metrics). We then calculated the average thickness of the layer of interest over the surface area of the Early Treatment Diabetic Retinopathy Study (ETDRS)-grid, excluding the fovea. For OAG cases, we used IOP, CDR, and OCT measurements of the affected eye. If both eyes were affected or unaffected, a random eye was selected. The date of death was obtained through notification by the local municipal administration and general practitioners office, or through notification by the nursing home and/or family (for participants that moved out of the Ommoord suburb after enrollment).

Assessment of covariates

Weight and height were measured at the research center. Body mass index (BMI) was calculated as weight in kilograms divided by the height in meters squared. Family history was assessed during home interviews. At the research center, blood pressure was measured at the right brachial artery with the participant in a sitting position. The mean of two consecutive measurements was used. Hypertension was defined as a resting blood pressure exceeding 140/90 mmHg or the use of blood pressure-lowering medication. Medication data were collected through home interviews and categorized using the Anatomical Therapeutical Chemical (ATC) coding system[9]. For this study, we collected data on blood pressure-lowering medications including diuretics, beta-blockers, calcium channel blockers, and renin-angiotensin-aldosterone system agents (ATC c02, c03, and c07-c09). Alcohol consumption (drinks per week), coffee consumption (cups per day), and tea consumption (cups per day) were assessed at baseline using food frequency questionnaires (FFQs) at-home interviews by trained dieticians as described in detail elsewhere[5]. Both Food Frequency Questionnaires that were used, were previously validated and showed reasonable to good estimates of nutrient intake[10-12]. All food items were assessed based on the frequency of consumption, the number of servings per day as well as on the preparation methods. Participants with unreliable dietary intake (total energy intake <500 kcal/day or >5000 kcal/day) were excluded. Total caffeine consumption was estimated using the median caffeine concentration in Dutch coffee and tea

according to the Dutch Center for Nutrition (45mg/100ml for coffee and 18.5mg/100ml for tea)[13]. Type 2 diabetes mellitus status was assessed by self-report during the home interview. Hypercholesterolemia was defined as a serum total cholesterol of 239mg/dL or more (to convert to millimoles per liter, multiply by 0.259)[14].

Supplementary references

1. Rich-Edwards JW, Corsano KA, Stampfer MJ. Test of the National Death Index and Equifax Nationwide Death Search. Am J Epidemiol. 1994 Dec 1;140(11):1016-9. doi: 10.1093/oxfordjournals.aje.a117191. PMID: 7985649.

2. Lindström S, Loomis S, Turman C, Huang H, Huang J, Aschard H, Chan AT, Choi H, Cornelis M, Curhan G, De Vivo I, Eliassen AH, Kraft P, et al. A comprehensive survey of genetic variation in 20,691 subjects from four large cohorts. PLoS One. 2017 Mar 16;12(3):e0173997. doi: 10.1371/journal.pone.0173997. PMID: 28301549; PMCID: PMC5354293.

3. Duffy DL, Zhu G, Li X, Sanna M, Iles MM, Jacobs LC, Evans DM, Yazar S, Beesley J, Law MH, Kraft P, Visconti A, Taylor JC, Liu F, Wright MJ, Henders AK, Bowdler L, Glass D, Ikram MA, Uitterlinden AG, Madden PA, Heath AC, Nelson EC, Green AC, Chanock S, Barrett JH, Brown MA, Hayward NK, MacGregor S, Sturm RA, Hewitt AW; Melanoma GWAS Consortium; Kayser M, Hunter DJ, Newton Bishop JA, Spector TD, Montgomery GW, Mackey DA, Smith GD, Nijsten TE, Bishop DT, Bataille V, Falchi M, Han J, Martin NG. Novel pleiotropic risk loci for melanoma and nevus density implicate multiple biological pathways. Nat Commun. 2018 Nov 14;9(1):4774. doi: 10.1038/s41467-018-06649-5. Erratum in: Nat Commun. 2019 Jan 14;10(1):299. PMID: 30429480; PMCID: PMC6235897.

4. O'Connell J, Gurdasani D, Delaneau O, et al. A general approach for haplotype phasing across the full spectrum of relatedness. PLoS Genet. 2014;10(4):e1004234. doi:10.1371/journal.pgen.1004234

5. Das S, Forer L, Schönherr S, et al. Next-generation genotype imputation service and methods. Nat Genet. 2016;48(10):1284-1287. doi:10.1038/ng.3656

6. Ikram MA, Brusselle G, Ghanbari M, Goedegebure A, Ikram MK, Kavousi M, Kieboom BCT, Klaver CCW, de Knegt RJ, Luik AI, Nijsten TEC, Peeters RP, van Rooij FJA, Stricker BH, Uitterlinden AG, Vernooij MW, Voortman T. Objectives, design and main findings until 2020 from the Rotterdam Study. Eur J Epidemiol. 2020 May;35(5):483-517. doi: 10.1007/s10654-020-00640-5. Epub 2020 May 4. PMID: 32367290; PMCID: PMC7250962.

7. Bengtsson B, Heijl A, Olsson J. Evaluation of a new threshold visual field strategy, SITA, in normal subjects. Swedish Interactive Thresholding Algorithm. Acta Ophthalmol Scand. 1998 Apr;76(2):165-9. doi: 10.1034/j.1600-0420.1998.760208.x. PMID: 9591946.

8. van der Valk R, Webers CA, Schouten JS, Zeegers MP, Hendrikse F, Prins MH. Intraocular pressure-lowering effects of all commonly used glaucoma drugs: a meta-analysis of randomized clinical trials. Ophthalmology. 2005 Jul;112(7):1177-85. doi: 10.1016/j.ophtha.2005.01.042. PMID: 15921747.

9. Cheng JW, Cheng SW, Gao LD, Lu GC, Wei RL. Intraocular pressure-lowering effects of commonly used fixed-combination drugs with timolol: a systematic review and meta-analysis. PLoS One.

2012;7(9):e45079. doi: 10.1371/journal.pone.0045079. Epub 2012 Sep 13. PMID: 23028770; PMCID: PMC3441590.

10. WHO. ATC/DDD Index 2022. https://www.whocc.no/atc_ddd_index/. Last updated 23.01.2023

11. Klipstein-Grobusch K, den Breeijen JH, Goldbohm RA, Geleijnse JM, Hofman A, Grobbee DE, Witteman JC. Dietary assessment in the elderly: validation of a semiquantitative food frequency questionnaire. Eur J Clin Nutr. 1998 Aug;52(8):588-96. doi: 10.1038/sj.ejcn.1600611. PMID: 9725660.

12. Feunekes GI, Van Staveren WA, De Vries JH, Burema J, Hautvast JG. Relative and biomarker-based validity of a food-frequency questionnaire estimating intake of fats and cholesterol. Am J Clin Nutr. 1993 Oct;58(4):489-96. doi: 10.1093/ajcn/58.4.489. PMID: 8379504.

13. Goldbohm RA, van den Brandt PA, Brants HA, van't Veer P, Al M, Sturmans F, Hermus RJ. Validation of a dietary questionnaire used in a large-scale prospective cohort study on diet and cancer. Eur J Clin Nutr. 1994 Apr;48(4):253-65. PMID: 8039485.

14. https://www.voedingscentrum.nl/factsheetcafeine. Accessed 06.04.2023

15. Expert Panel on Detection, Evaluation, and Treatment of High Blood Cholesterol in Adults. Executive Summary of The Third Report of The National Cholesterol Education Program (NCEP) Expert Panel on Detection, Evaluation, And Treatment of High Blood Cholesterol In Adults (Adult Treatment Panel III). JAMA. 2001 May 16;285(19):2486-97. doi: 10.1001/jama.285.19.2486. PMID: 11368702.

16. Craig JE, Han X, Qassim A, Hassall M, Cooke Bailey JN, Kinzy TG, Khawaja AP, An J, Marshall H, Gharahkhani P, Igo RP Jr, Graham SL, Healey PR, Ong JS, Zhou T, NEIGHBORHOOD consortium; UK Biobank Eye and Vision Consortium; MacGregor S, et al. Multitrait analysis of glaucoma identifies new risk loci and enables polygenic prediction of disease susceptibility and progression. Nat Genet. 2020 Feb;52(2):160-166. doi: 10.1038/s41588-019-0556-y. Epub 2020 Jan 20. PMID: 31959993; PMCID: PMC8056672.

17. Gharahkhani P, Jorgenson E, Hysi P, Khawaja AP, Pendergrass S, Han X, Ong JS, Hewitt AW, Segrè AV, Rouhana JM, Hamel AR, Igo RP Jr, Choquet H, Qassim A, NEIGHBORHOOD consortium; ANZRAG consortium; Biobank Japan project; FinnGen study; UK Biobank Eye and Vision Consortium; GIGA study group; 23 and Me Research Team; Wiggs JL, et al. Genome-wide meta-analysis identifies 127 open-angle glaucoma loci with consistent effect across ancestries. Nat Commun. 2021 Feb 24;12(1):1258. doi: 10.1038/s41467-020-20851-4. PMID: 33627673; PMCID: PMC7904932.

18. Han X, Gharahkhani P, Hamel AR, Ong JS, Rentería ME, Mehta P, Dong X, Pasutto F, Hammond C, Young TL, Hysi P, Lotery AJ, Jorgenson E, Choquet H, Hauser M, 23andMe Research Team; International Glaucoma Genetics Consortium; MacGregor S, et al. Large-scale multitrait genome-wide association analyses identify hundreds of glaucoma risk loci. Nat Genet. 2023 Jul;55(7):1116-1125. doi: 10.1038/s41588-023-01428-5. Epub 2023 Jun 29. PMID: 37386247; PMCID: PMC10335935
