# Supplementary material for: The Clinical Usefulness of a Glaucoma Polygenic Risk Score in 4 Population-Based European Ancestry Cohorts
Source: Ophthalmology. Author manuscript; Available in PMC 2025 Jun 27. (PMC12204775; doi:10.1016/j.ophtha.2024.08.005)
Supplement: Table S6 [file NIHMS2083589-supplement-Table_S6.pdf]

Supplementary Table S6. Comparison of the concordance (Harrell's C-statistic) of our polygenic risk score and previously published genetic risk scores (GRS), with corresponding 95% confidence intervals. Age, sex, IOP >25 mmHg, and family history were included in all models.

	Number of SNPs	C-index (US cohorts)	C-index (RS-I)	C-index (meta-analysis)
Craig et al, 2020[14]	2,673	0.77 (0.75, 0.79)	0.70 (0.65, 0.75)	0.76 (0.74, 0.78)
Gharahkhani et al, 2021[15]	127	0.77 (0.76, 0.79)	0.71 (0.67, 0.76)	0.76 (0.75, 0.78)
Han et al, 2023[16]	312	0.78 (0.76, 0.80)	0.73 (0.69, 0.78)	0.78 (0.76, 0.79)
Current study	144,020	0.82 (0.80, 0.84)	0.82 (0.78, 0.86)	0.82 (0.80, 0.84)

The GRS from Craig, et al. was calculated using the SNPs with optimized glaucoma risk prediction as reported by Craig et al, which was all SNPs with a p-value < 0.001, after LD-clumping with $R^2 = 0.1$ and p-value < 0.001 thresholds. The GRS from Gharahkhani and Han et al. were calculated using SNPs that were genome-wide significant ($p < 5 * 10^{-8}$).
