# Supplementary material for: The Clinical Usefulness of a Glaucoma Polygenic Risk Score in 4 Population-Based European Ancestry Cohorts
Source: Ophthalmology. Author manuscript; Available in PMC 2025 Jun 27. (PMC12204775; doi:10.1016/j.ophtha.2024.08.005)
Supplement: Table S7 [file NIHMS2083589-supplement-Table_S7.pdf]

**Supplementary Table S7.** Area under the receiver operating characteristic curve (AUC) with corresponding 95% confidence intervals based on predicted probabilities from logistic regression models		
	**AUC (US cohorts)**	**AUC (RS-I)**
Age + sex	0.63 (0.61, 0.64)	0.55 (0.50, 0.59)
Age + sex + IOP >25 mmHg + family history	0.75 (0.73, 0.77)	0.66 (0.61, 0.71)
Age + sex + IOP >25 mmHg + family history + PRS	0.82 (0.80, 0.84)	0.80 (0.76, 0.83)

IOP = intraocular pressure, PRS = polygenic risk score, US = United States, RS = Rotterdam Study
